# Supplementary material for: Anti-cancer effect of a novel photodynamic therapy using glucose-linked chlorin e6 conjugated trastuzumab for HER2-positive gastrointestinal cancers
Source: PLoS One. 2026 Apr 3;21(4):e0346567. doi: 10.1371/journal.pone.0346567 (PMC13048491; doi:10.1371/journal.pone.0346567)
Supplement: Attachment — Submitted filename: Response to Reviewers.docx [file pone.0346567.s002.docx]

Date: 09-Dec-2025

PONE-D-25-57729

Title: Anti-cancer effect of a novel photodynamic therapy using glucose-linked chlorin e6 conjugated trastuzumab for HER2-positive gastrointestinal cancers

Major revision

**Journal Requirements:**

1. Please ensure that your manuscript meets PLOS ONE's style requirements, including those for file naming.

I revised my manuscript, and confirmed that my revised manuscript meets PLOS ONE's style requirements.

2. We noticed you have some minor occurrence of overlapping text with the following previous publication(s), which needs to be addressed:

I cited our research group’s report (https://www.mdpi.com/2072-6694/11/5/636) to manuscript that was previously published. However, I can’t point out the overlapping text with the publication <https://www.mdpi.com/1422-0067/25/6/3233>. Although there may be some overlap in the methodologies, it is minimal.

3. Please include captions for your Supporting Information files at the end of your manuscript, and update any in-text citations to match accordingly. Please see our Supporting Information guidelines for more information: <http://journals.plos.org/plosone/s/supporting-information>.

I have no Supporting Information file.

4. If the reviewer comments include a recommendation to cite specific previously published works, please review and evaluate these publications to determine whether they are relevant and should be cited. There is no requirement to cite these works unless the editor has indicated otherwise.

There is no recommendation to cite specific previously published works.

**Reviewer’s Comments to Author:**

Reviewer: 1

This report relates to the use of a photosensitizing agent for treatment of GI cancer.

1) Line 34: if the selectivity of the photosensitizer is appropriate, there should be no damage to ‘normal mucosa’ because these cells should not be photosensitized.

Thank you for your comment. To the best of our knowledge, there is no photosensitizer that shows absolute and exclusive accumulation in tumor cells without any distribution to normal cells. Furthermore, while it is theoretically correct that super selective photosensitizers should not damage normal mucosa, drug distribution can be affected by inflammation, regenerative epithelium, and tumor heterogeneity in clinical cases. Therefore, improving selectivity remains an important clinical challenge. Tumor selectivity in photodynamic therapy remains a relative concept, and strategies to further enhance tumor-specific accumulation are still required.

2) If there is no selectivity for sites of neoplasia, a CO_2_ laser could be used for therapy.

Thank you for your comment. While CO₂ laser ablation may be feasible for superficial tumor removal, the extremely shallow penetration depth limits its usefulness for reducing advanced tumor volume in gastrointestinal cancers. Therefore, CO₂ laser therapy aimed at gastrointestinal tumor debulking is not commonly adopted in current clinical practice. In contrast, photodynamic approaches offer the potential to affect tumor tissue beyond the immediate surface.

3) It appears that the agent being used is thought to have insufficient targeting properties so it need to be conjugated to an antibody. There are many photosensitizing agents in use today that do not need to be conjugated to anything.

We appreciate your comment. Talaporfin sodium (TS) is currently the most widely used photosensitizer in clinical practice in Japan. Compared with the conventional agent porfimer sodium, TS exhibits enhanced tumor accumulation and a substantially shorter light-shielding period, which has led to its widespread clinical adoption in Japan. G-Ce6 is a novel photosensitizer that we synthesized and reported in 2018. *In vitro* photodynamic therapy using G-Ce6 demonstrated an anti-tumor effect that was approximately 9,000–34,000 times stronger than that of TS (These points were described in Reference 8. In addition, we have added an additional reference 11 (Osaki et al, Cancers, 2019.) related to G-Ce6 in the revised manuscript.). Taken together, these findings indicate that G-Ce6 is a highly potent photosensitizer with markedly enhanced cellular accumulation and photodynamic efficacy. The rationale for using an antibody-conjugated photosensitizer in this study was to introduce molecular targeting toward HER2-positive tumors. Our approach is intended as a complementary strategy for HER2-positive tumors, rather than a universal replacement for existing agents. Enhanced tumor accumulation compared with conventional agents provides several clinical advantages, including not only a reduction in damage to surrounding normal mucosa caused by scattered light, but also the feasibility of a shorter light-shielding period.

4) In vitro studies involved a 16 J/sq cm light dose which is very high for in vitro experiments. For most such studies, light doses in the range of 300-500 mJ/sq cm are used.

Thank you for your comment. We reviewed several publications on photodynamic therapy (PDT) using TS and found that, in *in vitro* experiments, irradiation is generally performed at the same order of magnitude as in our study, and none of the studies used irradiation doses in the mJ/cm² range. (7.5 J/cm^2^; Ezzaldeen et al, 2025, 10 J/cm^2^; Miki et al, 2013, 13 J/cm^2^; Akter et al, 2019, 0～10 J/cm^2^; Ichikawa et al, 2013). Therefore, we believe that this irradiation dose cannot be considered outside the standard range.

5) Details for the ROS assay indicate (line 120) that cells were irradiated, then exposed to DCFH-DA which is incorrect. DCFH-DA needs to be present during irradiation, since the ROS being formed are rapidly quenched. This error indicates that the authors are inexperienced in PDT technology.

Thank you for your comment. According to the protocol for H2DCFDA that we used (Invitrogen™), the recommended incubation time is 5–60 minutes. It is well known that it may cause dye–dye quenching when H2DCFDA is present in excess within cells. As you pointed out, we performed irradiation after co-incubating both the photosensitizers (G-Ce6 and G-Ce6-trastuzumab) and H2DCFDA for 60 minutes so that H2DCFDA would be present during irradiation; however, this approach did not yield satisfactory results. We consider that this may be due to the 60-minute incubation being insufficient for adequate cellular uptake of the photosensitizers.

An alternative approach—incubating cells with the photosensitizer first, washing, and then adding H2DCFDA in PBS before irradiation—was not performed, because the concentration of the photosensitizer used in this study (9.6n mol/L ; we apologize for the error in the initial version and have corrected it) was relatively low, and changing to PBS and incubating for longer time under PBS before irradiation was a concern as it could further dilute the photosensitizers.

Therefore, consistent with several previous reports (Huang et al, BMC cancer, 2019; Fatima et al, Journal of Advanced Research 2021), we administered H2DCFDA immediately after irradiation and evaluated ROS generation under these conditions.

6) What is the basis for the cell counting kit as an indicator of photokilling? The most unambiguous approach is to use a clonogenic assay which measures the ability of cells to form colonies. Anything else is only an approximation. Oleinick described a comparison between clonogenic assays and an alternative approach (Photochem Photobiol 2009) and found that the correlation was poor.

Thank you for your comment. We acknowledge that clonogenic assays are the gold standard for evaluating long-term reproductive cell death and are particularly appropriate for assessing cytotoxicity after prolonged drug exposure.

In this study, however, PDT involves a *single light irradiation*, and cytotoxic effects are typically evident within 24 hours after treatment. Therefore, for evaluating acute photokilling following a single PDT session, we consider the cell counting kit assay to be appropriate.

In contrast, clonogenic assays require long-term culture, during which additional confounding factors may influence colony formation. While clonogenic assays are valuable for long-term survival analysis, we believe that the cell counting kit assay is suitable for the aims of the present study.

7) Line 232 indicates a ‘specific wavelength of light’. All wavelengths of light are ‘specific’. What is needed is light at a wavelength that corresponds to an absorbance optimum of the photosensitizer, preferably one that can penetrate into tissues.

Thank you for your comment. We acknowledge that the term “specific” may cause misunderstanding. Accordingly, we have revised the expression to “optimal for the photosensitizer” to more accurately reflect our intention.

Summary: The ROS detection procedure should be carried out with the reagent present during irradiation. Any result that claims to detect photokilling should involve a clonogenic assay which detects the ability of cells to proliferate. It appears that the photosensitizer being used requires the presence of an antibody to provide adequate targeting. There are photosensitizers that do not have such a requirement. Does this agent represent a significant improvement over what is already available? It is true that the use of 654 nm light would represent an improvement over the 630 nm wavelength used with Photofrin.

Thank you for your valuable comments. We acknowledge that the presence of the ROS detection reagent during light irradiation is an important consideration. However, as described above, due to issues related to the concentration of the photosensitizer and the required incubation time, we addressed this concern by adding the ROS detection reagent H2DCFDA immediately after irradiation. In this study, our primary objective was to evaluate acute photokilling following a single PDT session using the cell counting kit assay. The newly developed photosensitizer G-Ce6-trastuzumab is designed to achieve high tumor accumulation through antibody-mediated targeting. The key features of this photosensitizer include the potential to reduce phototoxicity in non-tumor tissues by improving tumor specificity, as well as a reduction in the required drug dose. We consider these points to represent important advantages of this agent over existing photosensitizers.

Furthermore, the ability to use long-wavelength light at 654 nm offers a clear advantage in terms of improved tissue penetration compared with the 630 nm wavelength used for Photofrin, which we believe is of clinical relevance.

Taken together, compared with conventional photosensitizers, this novel photosensitizer G-Ce6-trastuzumab demonstrates clinical significance in terms of (i) enhanced tumor selectivity through targeting, (ii) reduced nonspecific phototoxicity, and (iii) improved tissue penetration enabled by long-wavelength excitation.
