# Supplementary material for: Anti-cancer effect of a novel photodynamic therapy using glucose-linked chlorin e6 conjugated trastuzumab for HER2-positive gastrointestinal cancers
Source: PLoS One. 2026 Apr 3;21(4):e0346567. doi: 10.1371/journal.pone.0346567 (PMC13048491; doi:10.1371/journal.pone.0346567)
Supplement: Attachment — Submitted filename: Response_to_Reviewers_auresp_2.docx [file pone.0346567.s003.docx]

Date: 09-Dec-2025

PONE-D-25-57729R1

Title: Anti-cancer effect of a novel photodynamic therapy using glucose-linked chlorin e6 conjugated trastuzumab for HER2-positive gastrointestinal cancers

**Journal Requirements:**

1. If the reviewer comments include a recommendation to cite specific previously published works, please review and evaluate these publications to determine whether they are relevant and should be cited. There is no requirement to cite these works unless the editor has indicated otherwise.

We have revised the manuscript by adding a relevant reference in response to the reviewer’s comment.

2. Please review your reference list to ensure that it is complete and correct. If you have cited papers that have been retracted, please include the rationale for doing so in the manuscript text, or remove these references and replace them with relevant current references. Any changes to the reference list should be mentioned in the rebuttal letter that accompanies your revised manuscript. If you need to cite a retracted article, indicate the article’s retracted status in the References list and also include a citation and full reference for the retraction notice.

We have confirmed that the reference list is complete and accurate.

**Additional Editor Comments:**

The reviewer still has concerns. You should use both PBS and medium for PDT, measure ROS both during and after light and not use "viability" for CC8-K assay.

We have addressed the reviewer’s comments regarding the rationale for not using culture medium during PDT and the measurement of ROS. In addition, the term “viability” has been revised to “cell survival rate at 24 hours after PDT.”

**Reviewers' comments:**

Reviewer's Responses to Questions

Comments to the Author

6. Review Comments to the Author

Please use the space provided to explain your answers to the questions above. You may also include additional comments for the author, including concerns about dual publication, research ethics, or publication ethics. (Please upload your review as an attachment if it exceeds 20,000 characters)

Reviewer #1:

**Looking sequentially through this report, why is ‘the aging population’ singled out (line 53)? Cancer can occur at any age**.

Thank you very much for your valuable comment.　As you pointed out, cancer can occur at any age. However, lung cancer and esophageal cancer—both of which are indications for photodynamic therapy (PDT) using talaporfin sodium—are generally considered to occur as results of the accumulation of genetic mutations caused by external factors such as smoking. Therefore, as the population ages, the absolute number of patients with cancer is increasing (Global cancer statistics 2022).　In addition, surgical resection and chemoradiotherapy, which are standard treatment options, are highly invasive. In elderly patients, these treatments may not be feasible due to limited tolerance or poor physical condition. Consequently, in the aging population, there is an increasing need for less invasive anticancer treatments. PDT, which is a minimally invasive treatment that does not require surgery or systemic chemotherapy, is therefore attracting considerable attention in aging populations. In our original manuscript, this point was insufficiently described. We have revised the manuscript accordingly, as shown below. We believe that this revision will improve the clarity and understanding for readers. We sincerely appreciate the reviewer’s insightful comment.

(Before)

The development of non-invasive treatment methods for cancer is desirable for the aging population. Photodynamic therapy (PDT), an anticancer therapy that uses photosensitizers (PSs) at optimal light irradiation wavelengths, has attracted considerable attention.

(After)

The development of non-invasive treatments for cancer is particularly desirable in aging populations. Although cancer can occur at any age, most of cancers are generally considered to arise from the accumulation of genetic mutations caused by genetic and environmental factors. Consequently, with population aging, the absolute number of patients with cancer continues to increase worldwide (Global Cancer Statistics 2022). Surgical resection and chemoradiotherapy, which are standard treatment options for cancer, are highly invasive and may not be feasible in elderly patients because of limited physiological reserve or poor general condition. Therefore, there is an increasing demand for less invasive anticancer therapies in aging societies. Photodynamic therapy (PDT), an anticancer treatment that utilizes photosensitizers (PSs) activated by light at optimal wavelengths and does not require surgery or systemic chemotherapy, has thus attracted considerable attention as a promising therapeutic option for elderly patients.

**Where is the data indicating ‘mucosal damage’ (line 57)?**

Thank you very much for this important and insightful comment. We agree that this is a highly important point. To address this issue, we present preclinical data obtained from normal canine esophagus (Horimatsu et al., 2012, PLOS ONE). This study evaluated the safety of Talaporfin Sodium based PDT applied to the esophageal wall in dogs prior to its clinical application to human esophageal cancers. The results demonstrated that, even in normal tissue, higher irradiation doses were associated with increased toxicity. The esophagus is a narrow, tubular organ, and precise confinement of light irradiation is inherently challenging. In addition, because the esophagus is anatomically adjacent to the heart, the irradiation field may be affected by cardiac motion. Depending on the depth of patient sedation, respiratory motion can also contribute to fluctuations in the irradiation site. Owing to these factors, it is extremely difficult to completely restrict light exposure exclusively to the tumor lesion in clinical practice. To minimize mucosal injury caused by scattered light, various technical refinements are employed in clinical practice, such as attaching dedicated devices to the tip of the endoscope (Yano et al., Journal of Clinical Medicine, Fig. 4). Nevertheless, injury to the surrounding normal mucosa may still occur. Esophageal stricture, which is a known adverse event of PDT for esophageal cancer, is considered at least partly attributable to such damage to normal esophageal mucosa. Regarding post-PDT esophageal strictures, several clinical data have been reported (Tanaka et al., Journal of Clinical Medicine, 2025; Horimatsu et al., Journal of Gastroenterology, 2025). Given the importance of this issue, we have added these references to the manuscript. We believe that their inclusion will be beneficial for readers. Thank you again for your valuable suggestion.

**The light dose needed for efficacy (line 113) is substantially higher than most reported uses of PDT in vitro.**

Thank you for your comment. We reviewed even more previously published studies than we had at the time of our response in R1.　We　reviewed several publications on photodynamic therapy (PDT) using TS and found that, in in vitro experiments, irradiation is generally performed at the same order of magnitude as in our study. (7.5 J/cm^2^, Ezzaldeen et al., 2025; 10 J/cm^2^, Miki et al., 2013; 13 J/cm^2^, Akter et al., 2019; 0～10 J/cm^2^; Ichikawa et al., 2013; 10 J/cm^2^, Yamashita et al., 2023; 6～13.5 J/cm^2^, Akter et al., 2019; 90 or 180 mJ/cm^2^, Kessel et al., 2017). Meanwhile, our review of the reports on G-Ce6-PDT indicated that relatively high irradiation doses have been used in some experimental settings. Indeed, light doses of 189 J/cm² (Huang et al., 2024) and 300 J/cm² (Tian et al., 2023) have been reported, suggesting our setting of irradiation is not uncommon in G-Ce6–based in vitro PDT studies. In experimental systems using lower irradiation doses, outcome evaluations are often performed several days after irradiation (e.g., 3 days post-irradiation), which may partly explain the use of lower light doses in those studies. Therefore, we believe that this irradiation dose not be outside the standard range. In addition, this report is based on G-Ce6. G-Ce6 has been newly synthesized by our research group (Nishie et al., 2018). We obtained the results under the same *in vitro* PDT conditions as those used in the previous study, and the current experimental conditions were set in accordance with those reports.

**Cells in PBS are in a non-physiologic state which can initiate death pathways. Why was culture medium not used?**

Thank you for this important comment. Phenol red contained in culture medium has major absorption peaks at approximately 415 and 560 nm. In *in vitro* PDT experiments, study designs are generally planned to avoid optical interference caused by phenol red. Therefore, cells are commonly washed with phosphate-buffered saline (PBS) to remove both the photosensitizer and phenol red prior to light irradiation. In cases where prolonged irradiation is required, phenol red–free medium may be used during irradiation. However, when the irradiation time is limited to several seconds to a few minutes, as in this study, conducting irradiation under PBS is generally accepted and commonly employed in *in vitro* PDT experiments.

**As pointed out before, ROS do not persist for any significant time after formation. This means that** **DCFH-DA needs to be present during irradiation, not after. Only a few long-lasting ROS, e.g., peroxides, will be present when irradiation ends.**

Thank you for your comment. We sincerely appreciate this very important and constructive feedback. Please note that some parts of the following response overlap with our previous reply. According to the protocol for H2DCFDA (Invitrogen™) used in this study, the recommended incubation time is 5–60 minutes. It is well known that excessive intracellular accumulation of H2DCFDA may cause dye–dye quenching; therefore, we avoided incubation times longer than 60 minutes. To ensure the presence of H2DCFDA during irradiation, we also examined a protocol in which cells were co-incubated with the photosensitizers (G-Ce6 and G-Ce6-trastuzumab) and H2DCFDA for 60 minutes prior to irradiation. However, this approach did not yield reproducible or satisfactory results. We consider that this may be due to insufficient cellular uptake of the photosensitizers during the 60-minute incubation period. As an alternative approach, cells could be incubated with the photosensitizer first, washed with PBS, and then exposed to H2DCFDA after replacement with PBS before irradiation. However, the concentration of the photosensitizer used in this study (9.6 nmol/L) was relatively low, and we were concerned that prolonged incubation under PBS conditions prior to irradiation could further dilute or promote leakage of the photosensitizer from the cells. For this reason, this approach was not adopted. Based on these considerations, and in line with several previous reports, we administered H2DCFDA immediately after irradiation. However, this method predominantly detects relatively long-lasting ROS, such as peroxides, and does not capture all ROS species generated by PDT. We have therefore explicitly described this limitation in the revised manuscript, as detailed below.

(addition to Discussion section)

This study has several limitations. In particular, the ROS detection method used in this study mainly detects relatively long-lived ROS (e.g., peroxides) and therefore does not comprehensively reflect all ROS species generated by PDT. DCFH-DA needs to be present during irradiation. However, the concentration of the photosensitizer used in this study (9.6 nmol/L) was relatively low, and we were concerned that prolonged incubation under PBS conditions containing H2DCFDA prior to irradiation could further dilute or promote leakage of the photosensitizer from the cells. Therefore, we administered H2DCFDA immediately after irradiation and evaluated ROS generation under these conditions.

**The accurate method for assessing viability is via clonogenic assays. If the authors want to use the CCK-8 kit, they need to report exactly what this kit measures, and it isn’t viability (as Oleinick showed in 2009).**

Thank you for your comment. As you pointed out, clonogenic assays represent the gold standard for evaluating long-term loss of proliferative capacity (reproductive cell death) and are particularly suitable for assessing cytotoxicity after prolonged drug exposure. We fully acknowledge this point. Oleinick et al. (2009) demonstrated that the results of clonogenic assays do not necessarily correlate with those of MTT assays; however, it is important to note that the MTT assay and the Cell Counting Kit-8 (CCK-8) assay used in the present study are based on different measurement principles.

In the present study, our primary objective was to evaluate short-term cellular responses following a single PDT treatment, and therefore we employed the CCK-8 assay. CCK-8 uses the novel tetrazolium salt WST-8, which is reduced via NADH generated by intracellular dehydrogenase activity to produce a water-soluble formazan. The absorbance of this formazan at 450 nm reflects cellular metabolic activity and is proportional to the number of viable cells. In contrast to the MTT assay, which mainly depends on mitochondrial enzyme activity and generates insoluble formazan crystals requiring consideration of reagent-associated cytotoxicity, WST-8 is water-soluble and has low intrinsic cytotoxicity, allowing more sensitive detection of short-term cytotoxic effects. For these reasons, we consider that the CCK-8 assay can detect acute cell damage more sensitively than the MTT assay.

The PDT protocol in this study was designed as a single light irradiation, and its cytotoxic effects typically become evident within 24 hours after treatment as acute photokilling. Accordingly, we selected the CCK-8 assay rather than clonogenic or MTT assays to assess these acute effects.

Nevertheless, as shown by Oleinick et al. (2009), delayed cell death mediated by slower mechanisms such as autophagy may occur at later time points following PDT. We have therefore described this limitation in the revised manuscript. In addition, to ensure accurate interpretation of the results, we have replaced the term “viability” with the more precise expression “cell survival rate at 24 hours after PDT” throughout the manuscript.

(addition to Discussion section)

Another limitation of this study is that slower mechanisms of cell death, such as autophagy (Oleinick et al), were not investigated. Nevertheless, because the PDT protocol was designed as a single light irradiation and cytotoxic effects are generally apparent within 24 hours as acute photokilling, the CCK-8 assay, that uses WST-8 and can detect acute cell damage more sensitively than the MTT assay, was considered appropriate for evaluating acute cell death in this study.

Summary: lacking an appropriate assay for ROS formation, there is no accurate estimate of formation of these species. The word ‘viability’ is incorrectly used in several places. With no pertinent assay for viability (clonogenics), this term cannot be used. The authors could report what they have found but need to be accurate in claims for what the results indicate.
