# Supplementary material for: Anti-cancer effect of a novel photodynamic therapy using glucose-linked chlorin e6 conjugated trastuzumab for HER2-positive gastrointestinal cancers
Source: PLoS One. 2026 Apr 3;21(4):e0346567. doi: 10.1371/journal.pone.0346567 (PMC13048491; doi:10.1371/journal.pone.0346567)
Supplement: Attachment — Submitted filename: Response_to_Reviewers_auresp_3.docx [file pone.0346567.s004.docx]

Date: 01-Mar-2026

PONE-D-25-57729R2

Title: Anti-cancer effect of a novel photodynamic therapy using glucose-linked chlorin e6 conjugated trastuzumab for HER2-positive gastrointestinal cancers

**Dear Editor and Reviewers,**

We sincerely appreciate the opportunity to resubmit our revised manuscript and are grateful for the careful evaluation of our work. We have thoroughly revised the manuscript in response to all reviewer comments and have clarified methodological details and limitations to ensure scientific accuracy and transparency.

Below we provide a point-by-point response.

**Reviewer #1**

**1. “Aging population” statement**

We thank the reviewer for this important comment.

We agree that cancer can occur at any age. We have revised the Introduction to clarify that while cancer affects all age groups, the absolute number of cases increases with population aging due to cumulative genetic and environmental factors. We also clarified the clinical rationale for minimally invasive treatments in elderly populations.

The revised text has been incorporated accordingly.

**2. Evidence for mucosal damage**

We appreciate this insightful comment.

We have now added relevant preclinical and clinical references demonstrating that higher irradiation doses may cause damage to surrounding esophageal mucosa and contribute to post-PDT strictures. These include data from canine models and recent clinical reports.

The manuscript has been revised to include these references and contextual clarification.

**3. Light dose used in vitro**

We thank the reviewer for raising this concern.

We have re-examined the literature and confirmed that irradiation doses of similar magnitude have been reported in TS- and G-Ce6–based in vitro PDT studies. In addition, G-Ce6–based systems have occasionally required relatively high light doses.

We have clarified this point in the revised manuscript and included additional supporting references.

**4. Use of PBS instead of culture medium during irradiation**

We appreciate this important methodological question.

Phenol red in culture medium absorbs at wavelengths overlapping with PDT excitation spectra and may interfere with irradiation. Therefore, washing with PBS before irradiation is a widely accepted approach in short-duration in vitro PDT experiments.

We have clarified this rationale in the revised manuscript.

**5. ROS measurement methodology**

We sincerely thank the reviewer for this highly important comment.

We fully acknowledge that DCFH-DA ideally should be present during irradiation to capture short-lived ROS.

However:

- The photosensitizer concentration used (9.6 nmol/L) was relatively low.
- Prolonged pre-irradiation incubation under PBS conditions risked dilution or leakage.
- Co-incubation approaches did not produce reproducible results.

Therefore, H2DCFDA was administered immediately after irradiation, which primarily detects relatively long-lived ROS (e.g., peroxides).

Importantly, we have now:

- Explicitly stated this limitation in the Discussion
- Clarified that our method does not comprehensively quantify all ROS species
- Modified the wording to avoid overinterpretation

We agree that this method does not provide a complete quantitative estimate of all ROS species generated during PDT.

**6. Use of the term “viability” and lack of clonogenic assay**

We thank the reviewer for this critical comment.

We fully agree that clonogenic assays represent the gold standard for assessing long-term reproductive cell death. However, our study aimed to evaluate short-term cytotoxic effects 24 hours after a single PDT exposure.

Therefore:

- We used the CCK-8 assay (WST-8 based), which measures intracellular dehydrogenase activity and reflects metabolic activity proportional to surviving cell number.
- We have replaced all instances of “viability” with “cell survival rate at 24 hours after PDT.”
- We have clarified what CCK-8 measures.
- We have explicitly acknowledged that clonogenic survival was not assessed and that slower cell death mechanisms were not evaluated.

We have carefully revised the manuscript to ensure terminological precision and avoid overstatement.

**Reviewer Summary Comment**

The reviewer expressed concern that without an appropriate ROS assay and clonogenic assay, the claims may not be fully supported.

We appreciate this important observation.

In response:

- We have clarified the limitations of ROS detection.
- We have corrected terminology regarding “viability.”
- We have carefully revised the Discussion to ensure that conclusions are restricted to the data obtained.
- We have avoided claims implying comprehensive ROS quantification or long-term reproductive death.

We believe the revised manuscript now accurately reflects the scope and limitations of the data presented.

We sincerely thank the reviewers for their constructive and scientifically rigorous comments. We believe that the revisions have substantially improved the clarity, transparency, and scientific precision of the manuscript.

Sincerely,

Mamoru Tanaka, M.D., Ph.D.

On behalf of all authors
